# Rapid processing window development of Mo-Si-B alloy for electron beam powder bed fusion

**DOI:** 10.1007/s40964-025-01119-z

**Published:** 2025-04-28

**Authors:** Yong Chen, Jonas Böhm, Benjamin Wahlmann, Manja Krüger, Carolin Körner

**Affiliations:** 1 Center of Advanced Materials and Processes, Friedrich-Alexander University Erlangen-Nürnberg, Dr.-Mack-Straße 81, 90762 Fürth, Germany; 2 Department of Materials Science, Chair of Materials Science and Technology for Metals, Friedrich-Alexander University Erlangen-Nürnberg, Martensstrasse 5, 91058 Erlangen, Germany; 3 Institute of Materials and Joining Technology, Otto-Von-Guericke-University Magdeburg, Universitätsplatz 2, 39106 Magdeburg, Germany

**Keywords:** Mo-Si-B alloy, Electron beam powder bed fusion (PBF-EB), Process monitoring, Processing window, Thermal modeling

## Abstract

The multiphase alloy Mo-9Si-8B (at.%) exhibits high oxidation, creep, and fracture resistance at high temperatures. With a melting point of about 2360 °C, it is a promising material for ultra-high temperature applications in turbine engines. However, Mo-9Si-8B (at.%) is difficult to process by traditional manufacturing methods due to its brittleness. Additive manufacturing offers a solution by enabling the production of complex near-net-shape bulk materials (e.g., turbine blades) in a single step. In this study, electron beam powder bed fusion (PBF-EB), which is characterized by extremely high local processing temperatures and associated high powder bed temperatures (i.e., above the brittle-to-ductile transition temperature of the material), was employed to process this Mo-Si-B alloy. The processing window was rapidly developed for the first time using novel strategies that combine high-throughput thermal modeling to predict the melt pool dimensions with in situ electron-optical imaging. High-density bulk Mo-9Si-8B (at.%) samples were successfully fabricated according to the established processing window, and the typical microstructure and phase composition of the as-built samples were analyzed. This novel approach significantly reduces the effort required to generate processing windows, making it highly viable for developing stable processing conditions for new materials in PBF-EB.

## Introduction

The continuing need to enhance the efficiency of gas turbine engines in aerospace and power generation, which requires operation at higher temperatures, has spurred the search for new ultrahigh-temperature structural materials [1, 2]. With its high melting temperature, molybdenum (Mo) is a promising candidate for high-temperature applications [3]. However, its application temperature in oxidative environments is restricted at or above ∼ 500 °C due to the rapid formation and subsequent sublimation of a MoO_3_ oxide scale [4]. With the addition of silicon (Si) and boron (B), the oxidation resistance was enhanced through the formation of a protective SiO_2_ scale [5]. Simultaneously, intermetallic compounds such as Mo_3_Si and Mo_5_SiB_2_, which are formed in this system, serve as high-temperature reinforcing phases and exhibit excellent oxidation and creep resistance, as well as good strength retention at high temperatures [6–8]. Hence, the Mo-Si-B refractory silicides have attracted significant research interest [3, 9]. However, these intermetallic compounds are extremely brittle, resulting in poor toughness and challenging processing [10, 11]. To address these issues, multiphase alloys have been developed that combine ductile and intermetallic phases, including the Mo solid solution phase (Mo_SS_) and the silicides Mo_5_SiB_2_ and Mo_3_Si, exhibiting optimal oxidation, creep, and fracture resistance [1, 3, 12]. So far, the ingot metallurgy method via repeated arc-melting and solidification has been the most utilized processing method in the study of Mo-Si-B alloys, owing to its relative ease of processing [13, 14]. In addition, powder metallurgy methods have been developed to process these alloys using solid-state reactions [15–17]. Nevertheless, these methods have inherent limitations such as restricted design flexibility and complicated processes. They require significant effort, including time- and cost-intensive processing, to achieve the desired microstructures. This processing involves prolonged high-temperature treatments, the application of pressure, or a combination of both [18, 19].

Additive manufacturing (AM) techniques offer unrivaled benefits in terms of design freedom and a reduction in fabrication steps, enabling the production of complex near-net-shape bulk materials (e.g., turbine blades) in a single step [20]. Directed energy deposition (DED) and powder bed fusion (PBF) are the most widespread AM processes for producing high-value, complex structural parts from key aero-engine materials [21, 22]. Schmelzer et al. demonstrated the feasibility of using DED for additive manufacturing with pre-alloyed near-eutectic Mo-13.5Si-7.5B (at.%) powders [23]. Following this, Mo-9Si-8B (at.%) alloys, which have a higher melting point of ~ 2360 °C compared to the near-eutectic alloy (~ 2000 °C), were fabricated by DED, as reported by Krüger et al. [24]. While DED offers a larger build envelope and higher deposition rate than PBF, it has limitations in fabricating finer geometries and hollow structures. PBF is a process in which powder layers are selectively melted using a high energy heat source, producing parts layer by layer with almost unlimited geometrical freedom, enabling the construction of complex features, hollow cooling passages, and high-precision components [20, 21]. PBF methods are classified based on the heat source used: laser powder bed fusion (PBF-LB) and electron beam powder bed fusion (PBF-EB). Fichtner et al. utilized an inductive preheating system to achieve the required high substrate temperatures of 1200 °C during PBF-LB, which enabled the fabrication of a high-density Mo-Si-B alloy using near-eutectic Mo-16.5Si-7.5B (at.%) powder [25]. Given the high brittle-to-ductile transition temperature (BDTT) of the Mo-Si-B alloy (i.e., exceeding 1000 °C), producing crack-free samples necessitates high processing temperatures. Contrastingly, Mo-Si-B-TiC alloys fabricated via PBF-LB without preheating exhibit extensive cracking across their microstructure [26].

PBF-EB (also known as electron beam melting, EBM), which is characterized by high energy input and absorption, enables extremely high local processing and powder bed temperatures, making it a promising approaches for producing high-performance component [20]. The high powder bed temperatures in PBF-EB (i.e., above the BDTT of the material), combined with a vacuum environment, reduce thermal stress and cracking risk, making it a promising technology for producing refractory materials with complex geometries. Higashi et al. successfully fabricated crack-free MoSiBTiC alloys using PBF-EB, demonstrating the versatility and potential of this technology in processing refractory materials with high BDTT [27]. So far, the process development of new materials using PBF-EB has largely relied on trial-and-error methods, which are inherently costly and time-consuming. With the advent of reliable in situ process observation using electron-optical imaging (ELO), it has become possible to identify and classify sample surfaces immediately. Arnold et al. utilized ELO in situ imaging to monitor and predict manufacturing precision in PBF-EB, and demonstrated that ELO imaging accurately captures the molten layers [28]. Furthermore, ELO imaging can reliably detect major defects (e.g., pores, cracks) even under demanding process conditions, directly assessing the quality of the resulting component [29, 30]. This approach significantly reduces the time required for developing a processing window by applying only a limited number of representative layers for each parameter combination, eliminating the need for metallographic preparation. Pobel et al. reported that by utilizing in situ ELO monitoring, processing windows can be determined with sufficient accuracy, and the process window development time is substantially reduced from weeks or even months to just a single build job of several hours [31]. Despite the acceleration of parameter evaluation with ELO, the number of necessary experimental trials remains substantial for qualifying new materials due to the need for a detailed mapping of the processing window boundaries. Numerical simulations of the melting step can further accelerate the development of process parameters by predicting consolidation and persistence boundaries that guide the selection of appropriate parameters [32].

To the best knowledge of the authors, there are no reports available in the scientific literature on the process development of Mo-Si-B alloy using PBF-EB. The present work represents a pioneering effort in the rapid development of processing windows for the fabrication of dense Mo-Si-B alloys via PBF-EB. High-throughput thermal modeling is combined with in situ electron-optical imaging (ELO), delivering highly accurate results while significantly reducing the required experimental effort to just a single build job. Specimens of high-density Mo-9Si-8B (at.%) bulk material, featuring microstructural homogeneity, were fabricated using stable melting conditions according to these established parameters.

## Material and methods

Gas-atomized pre-alloyed Mo-9Si-8B (at.%) powder (GfE GmbH, Nürnberg, Germany) was utilized for PBF-EB in this work. The powder was sieved and recycled after each build. Its oxygen content was measured using a carrier gas hot extraction method with an oxygen–nitrogen analyzer (EMGA-920, HORIBA Ltd, Kyoto, Japan). Stainless steel 1.4571 (AISI 316 Ti) served as the substrate for the build process. The particle morphology of the Mo-9Si-8B (at.%) powder was investigated utilizing a scanning electron microscope (SEM, NanoLab 600 DualBeam, FEI—Quanta 450, USA). The cross-sectional microstructure and element distribution of the powders were analyzed using electron probe microanalysis (EPMA, JXA 8100, Jeol, Tokyo, Japan). The particle size distribution was measured by means of laser diffraction granulometry (LDG, Mastersizer 3000, Malvern GmbH, Kassel, Germany).

The processing window was predicted through high-throughput thermal simulations using a semi-analytical temperature model, derived from Fourier’s heat conduction equation [33]. The high computational throughput was achieved by neglecting physical effects such as fluid convection, latent heat release, thermal radiation and vaporization. Despite this simplified approach, the heat conduction-based model effectively delivered accurate melt pool geometry predictions in prior studies [34, 35]. Moreover, the model was implemented in Python using NumPy and mpi4py for efficient parallel evaluation. A more detailed description of the process window simulation is given by Breuning et al. [32]. The required material properties—specifically mass density, specific heat capacity, thermal conductivity, and liquidus temperature—were assumed to be constant and are summarized in Table 1. Within the scope of this study, the process window is defined in terms of area energy (*E*_A_ see Eq. (1)) and lateral velocity (*v*_lat_, see Eq. (2)), i.e., the velocity in the lateral direction over the course of the hatch, as follows:

1 $${\text{E}}_{\text{A}}\text{ = }\frac{{P}_{\text{beam}}}{{v}_{\text{beam}}\cdot {l}_{\text{o}}},$$

2 $${\text{v}}_{\text{lat}}\text{ = }\frac{{v}_{\text{beam}}\cdot {l}_{\text{o}}}{{l}_{\text{cube}}},$$

Here, *P*_beam_ denotes the beam power, *v*_beam_ the scanning velocity, *l*_o_ the line offset, and *l*_cube_ the edge length of the produced cuboids. The area energy and the lateral velocity were defined by varying *P*_beam_ and *v*_beam_, which ranged from 300 to 6300 W and 1.5–9 m/s, respectively. The line offset *l*_*o*_, the cube length *l*_cube_, and the build temperature *T*_build_ were kept constant. Their values, as well as the electron beam diameter and the layer thickness, are summarized in Table 2. They were kept consistent throughout the simulations and the subsequent experiments.

**Table 1:** Material parameters for Mo-9Si-B (at.%) as used in the semi-analytical thermal model

Parameter	Value	References
Mass density *ρ*_m_ [g/cm^3^]	9.58	[37]
Liquidus temperature *T*_lid_ [°C]	2400	[37]
Specific heat capacity *C*_P_ [J/(kg·K)]	360	[38]
Thermal conductivity *κ* [W/(m·K)]	50	[39]

**Table 2:** Fixed process parameters for PBF-EB of Mo-9Si-8B (at.%)

Build temperature *T*_build_ [°C]	~ 1000
Line-offset *l*_*0*_ [µm]	100
Size of cuboid samples (*l*_cube_ × *l*_cube_ × *h*_cube_) [mm^3^]	15 × 15 × 15
Beam diameter (full -width half power) [µm]	300
Layer thickness [µm]	50

For numerous combinations of *E*_A_ and *v*_lat_, the melt pool shape and depth were calculated and analyzed. Hereby, two boundaries for the processing window were mapped: the consolidation boundary at low energy input representing the minimum required energy input for effective consolidation of the powder, and the persistence boundary at high energy input, where fluid flow in the melt pool leads to an uneven surface structure. Within the scope of this study, sufficient connection was assumed when the melt pool depth exceeded the effective layer thickness. From the nominal layer thickness of 50 μm and a powder bulk density of 50%, the effective layer thickness could be calculated as 100 μm. Furthermore, the persistent melt pool was defined as a condition where the melt pool remains liquid when the beam returns, i.e., when the melt pool lifetime exceeds the beam return time [34]. In a persistent melt pool, the Marangoni effect—resulting from the significant temperature gradient between the center and the tail of the melt pool—combined with vapor recoil pressure and electron beam agitation, induces high-velocity liquid flow, which tends to form bumps, resulting in an uneven surface [36]. Thus, the persistence boundary indicates the upper energy input limit for achieving an ideal surface quality.

The PBF-EB processes were performed on a Freemelt One (Freemelt AB, Mölndal, Sweden), a freely programmable PBF-EB machine, which is equipped with an in-house developed ELO system capable of recording images during the process. Compared to the standard PBF-EB process, which involves a cycle of applying a new powder layer, heating, melting, and lowering the platform, an additional image acquisition step is included after the melting step in the ELO approach to enable layer-wise evaluation of the melted surface. ELO images were recorded by four detectors, with a beam current of 1 mA and a spatial resolution of 120 μm/pixel. Topographical information of the build surface was harvested via differencing images obtained from opposite detectors. These difference images were immediately used to classify the surfaces of the specimens as porous, good (dense and uniform), or uneven, thereby eliminating the need for metallographic preparation or further optical microscopy analysis of the as-built samples.

In this work, the temperature of the bottom side of the substrate, i.e., the build temperature, was measured using a thermocouple. The build temperature was maintained at ~ 1000 °C during the whole PBF-EB process. Columnar supports were built on the substrate prior to hatching the specimens. Following this setup, nine square melting areas, each measuring 15 × 15 mm^2^ and spaced 5 mm apart, were hatched in a single layer, featuring various combinations of *E*_A_ and *v*_lat_. Furthermore, a transition parameter was utilized to bridge the gap between support construction and hatching, ensuring a smooth transition. All areas were melted using a snake hatching pattern, with the hatching direction rotated by 90 degrees after each layer. In each cube, one parameter set of *E*_A_ and *v*_lat_ was employed for building 2.5 mm of the specimen. Following this, the surface quality was analyzed using ELO imaging. The height of 2.5 mm was empirically chosen to avoid any influence of the underlying parameter sets on the observed surface morphology. This approach enables the testing of multiple parameter sets—in this case up to 54—within a single build job, thus allowing for the determination of a complete processing window in a relatively short period.

Specimens measuring ~ 15 × 15 × 15 mm^3^ were fabricated using stable melting parameters producing dense samples according to the established processing window. Here, a constant *E*_A_ was maintained while varying the *v*_lat_. For microstructure characterization, the cuboids were first cut parallel to the build direction using wire erosion, followed by grinding and polishing. The prepared cross-sections were then investigated through an optical microscope (AXIO Imager M1 m, Carl Zeiss Jena GmbH, Germany). The area fraction of the porosity was measured on binarized images of the cross-sections, and the relative density was calculated. Furthermore, an SEM (Helios NanoLab600, FEI, USA) equipped with an electron back-scattered diffraction (EBSD) detector was employed to investigate the phase composition of the as-built samples.

## Results and discussion

**Fig. 1:**
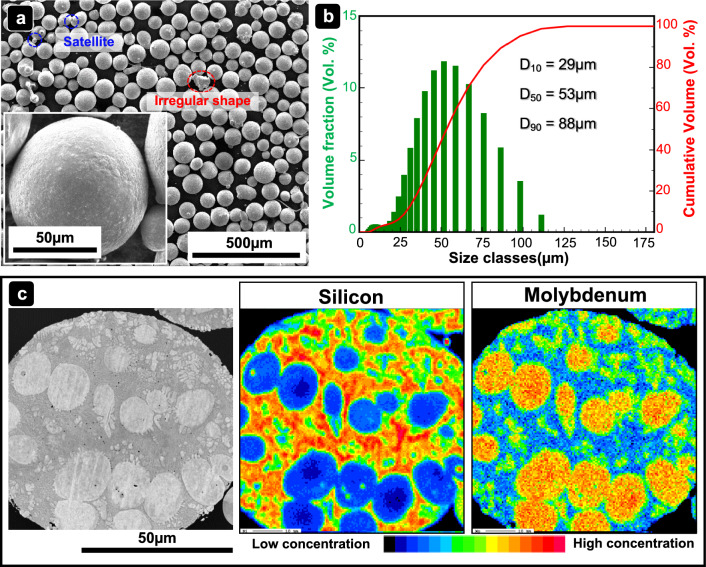
**a** SEM images of Mo-9Si-8B (at.%) powder particles at different magnifications. **b** Particle size distribution of the Mo-9Si-8B (at.%) powder determined by means of laser diffraction granulometry (LDG). **c** SEM image and corresponding electron probe microanalysis (EPMA) element mapping images of the cross-section of Mo-9Si-8B (at.%) powder

Figure 1a shows the particle morphology of the pre-alloyed Mo-9Si-8B (at.%) powder, predominantly comprising highly spherical particles. Irregularly shaped particles are highlighted within a red dotted circle, and satellite particles are indicated by a blue dotted circle. In addition, the high-magnification SEM image insert in Fig. 1a reveals a relatively rough surface of the particles. Figure 1b displays the particle size distribution of the Mo-9Si-8B (at.%) powder, which ranges widely from 5 to 110 µm, with a median size D_50_ of 53 µm. Figure 1c presents an enlarged cross-sectional view of an individual powder particle, showing primarily solidified molybdenum solid solution (Mo_SS_) in cellular and dendritic structures, surrounded by a matrix with high silicon content.

**Fig. 2:**
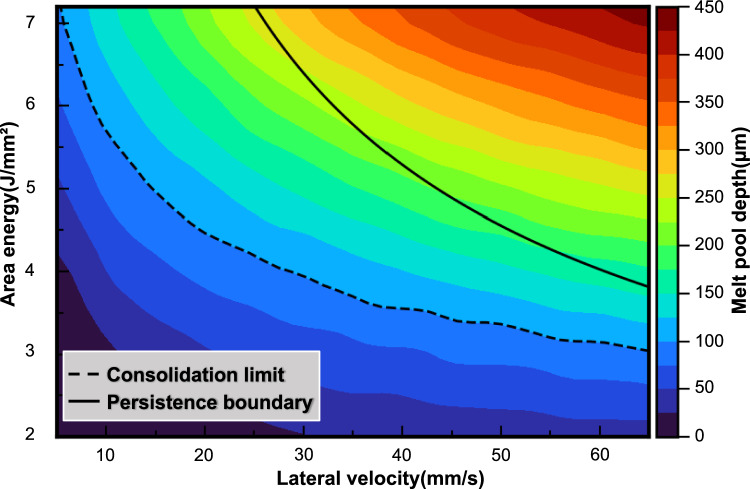
Calculated melt pool depths of Mo-9Si-8B (at.%) alloy via PBF-EB with varying combinations of area energies (*E*_A_) and lateral velocities (*v*_lat_). The derived processing window boundaries are indicated

Figure 2 illustrates the process boundaries for the PBF-EB processing of the Mo-9Si-8B (at.%) alloy, derived from process parameter-melt pool relationships obtained through high-throughput thermal modeling calculations. The consolidation boundary marks the lower bound of the processing map, ensuring sufficient connection between subsequent layers. The melt pool depth increases with higher *E*_A_ at a constant *v*_lat_, and with increased *v*_lat_ at a constant *E*_A_ due to the cumulative heating effect from previous scanning lines. Consequently, the consolidation boundary shifts towards lower *E*_A_ with higher *v*_lat_. As the energy input increases further, the melt pool transitions to a persistent state, causing fluid flow that leads to an uneven surface structure. Thus, the persistence boundary indicates the upper energy input limit for achieving an ideal surface quality. Therefore, the processing window for the Mo-Si-B alloy is preliminarily identified by the positions of the consolidation boundary and persistence boundary as predicted by the high-throughput thermal calculations.

**Fig. 3:**
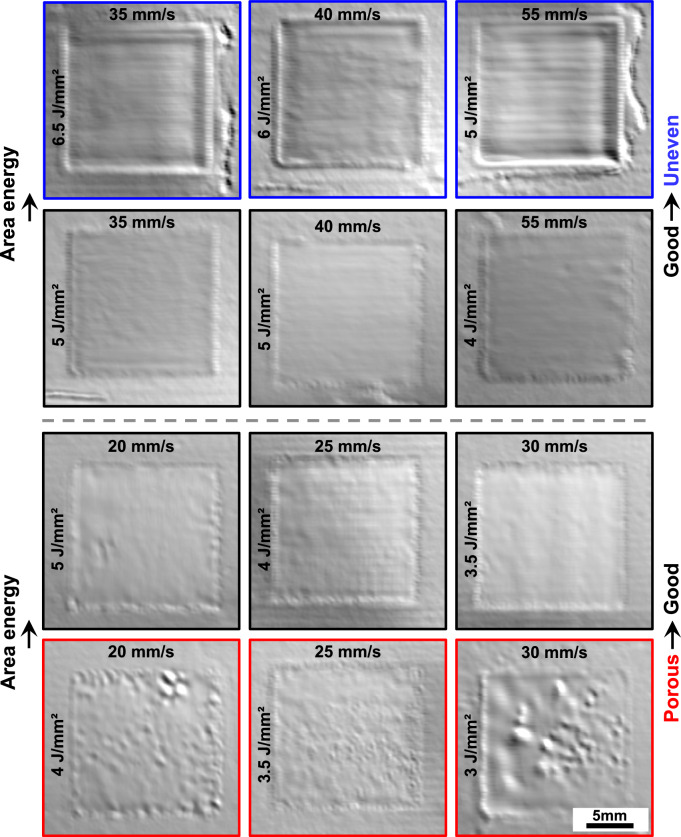
Electron-optical (ELO) difference images showing the surface morphology of Mo-9Si-8B (at.%) parts built by PBF-EB with varying combinations of area energies (*E*_A_) and lateral velocities (*v*_lat_). Surface classifications are marked as porous (red), good (black), and uneven (blue)

Figure 3 presents the exemplary ELO images classifying the surface morphologies of Mo-9Si-8B (at.%) specimens, highlighted with colors to distinguish between porous (red), good (black), and uneven (blue) surfaces. A clear transformation in surface morphology with increasing area energy is shown: from porous to good (below the gray dashed line), and from good to uneven (above the gray dashed line). Specifically, as the *E*_A_ increases from 4 J/mm^2^ to 5 J/mm^2^ at a constant *v*_lat_ of 20 mm/s, the surface morphology strongly improves in quality. Moreover, at a higher *v*_lat_ of 30 mm/s and a lower ***E***_**A**_ of 3.5 J/mm^2^, a planar surface morphology emerges, aligning with the consolidation boundary trends predicted by high-throughput thermal modeling. In addition, within the high-energy section of the process window, the transition of surface morphology from good to uneven aligns well with the trends of the predicted persistence boundary, characterized by a shift toward lower *E*_A_ at higher *v*_lat_. The complete processing window for the Mo-9Si-8B (at%) alloy via PBF-EB has been established through the ELO-based classification of all parameter sets.

**Fig. 4:**
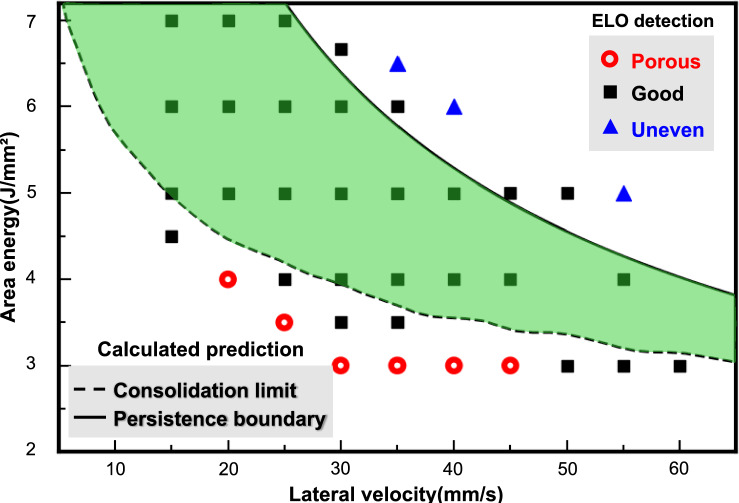
Processing window of Mo-9Si-8B (at.%) using PBF-EB, derived from a combination of numerical simulations and ELO monitoring approaches

The processing window for Mo-9Si-8B (at.%) alloy fabricated via PBF-EB is illustrated in Fig. 4. Derived from the ELO approach in a single build job, the processing window encompasses 38 data points, each with varying *E*_A_ and *v*_lat_. Meanwhile, the green area, which is defined by the consolidation limit and persistence boundary from high-throughput thermal modeling calculations, depicts the estimation of a stable processing region. The results from both methods show a high level of agreement. From these results, the processing window of Mo-9Si-8B (at.%) for the given process settings is plotted as a function of lateral velocity and area energy. A broader process window opens at lower lateral velocity, narrowing at higher lateral velocity. The integration of numerical simulations with ELO monitoring techniques enables the prediction of the upper and lower bounds of the processing window, thereby accelerating the identification of optimal process parameters. Furthermore, the use of in situ ELO monitoring eliminates the need for cumbersome sample preparation and analysis. This holistic strategy significantly saves time and cuts costs, making it an exceptionally efficient solution for developing new materials through additive manufacturing.

**Fig. 5:**
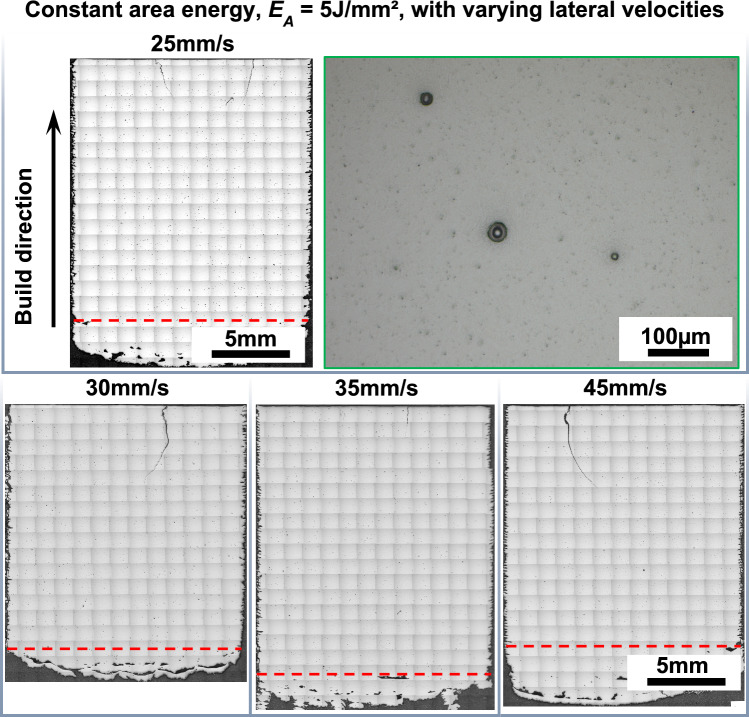
Overview of optical microscope images along the building direction of Mo-9Si-8B (at.%) processed via PBF-EB with varying lateral velocities with a constant area energy of 5 J/mm^2^. Inset details highlight the morphology of pores. The red dashed line represents the commencement of the hatching parameter

Cuboidal specimens, measuring 15 × 15 mm^2^ at the base and over 15 mm in height, were fabricated at varying *v*_lat_ and a constant *E*_A_ of 5 J/mm^2^, in accordance with the established processing window. Overview of optical micrographs of the sample cross-sections is shown in Fig. 5. Cracks extending from the top surface into the interior of the samples are observed. ELO imaging, which has been validated to reliably document surface cracks over extended building periods [30], did not detect these cracks during the building process. This suggests that the cracks did not form during the building process but afterwards during the cooldown phase of the build. The top surface of the sample undergoes rapid cooling due to the lack of insulation from the powder bed, resulting in a significant temperature gradient compared to the interior material. Cracks were initiated due to the thermal stresses induced by a significant temperature gradient, and the inherently high BDTT (~ 1000 °C) of the Mo-Si-B alloy [12]. Excluding crack defects in the upper part and the transition zone at the bottom, the relative density of these samples is over 99.7%. These could be prevented by reducing the temperature gradient on the top surface through layered powder application and controlled heating [40]. We aim to improve these issues in future studies. The presence of oxygen in the raw powder, as indicated by oxygen measurements, suggests that the observed pores, which are perfectly round and smaller than 50 microns, are likely caused by residual gas being trapped at the solid–liquid interface during rapid solidification and unable to escape.

**Fig. 6:**
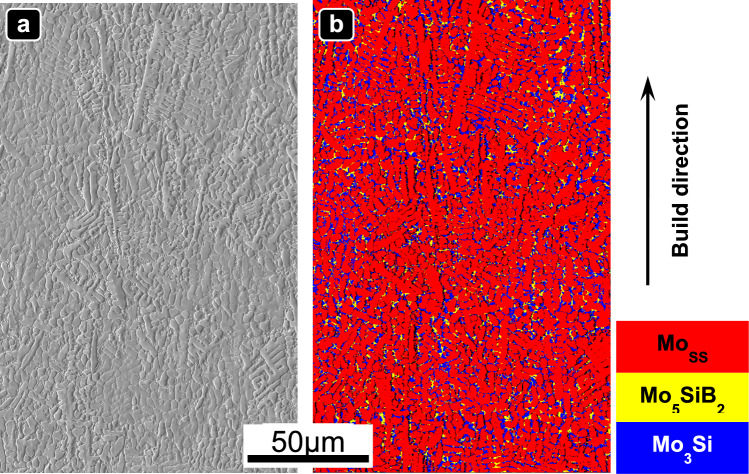
**a** SE image of the as-built Mo-9Si-8B (at.%) sample with an area energy of 5 J/mm^2^ and a lateral velocity of 45 mm/s. **b** EBSD image showing the color-coded phases: red—Mo_SS_, yellow—Mo_5_SiB_2_, blue—Mo_3_Si

EBSD analysis was performed to confirm the phase composition of the specimens. Figure 6 illustrates the microstructure of the PBF-EB-processed Mo-9Si-8B (at.%) alloy, consisting of three phases: Mo_SS_, Mo_5_SiB_2_ (tetragonal D8_*l*_, T2 phase), and Mo_3_Si (cubic A15 structure, A15 phase). A typical dendritic structure, characterized by coarse dendrite stems of the Mo_SS_ phase and fine interdendritic Mo_5_SiB_2_ and Mo_3_Si phases, was obtained. In contrast, the microstructure of the raw powder features Mo_SS_ phases with large spherical and finer dendritic structures embedded within a silicon-rich matrix (Fig. 1c). This significant transformation in microstructure highlights the profound impact of PBF-EB processing. Moreover, Krüger et al. have demonstrated that the proportions and morphologies of phases significantly influence the mechanical properties of the Mo-Si-B alloy [12]. The ideal microstructure of Mo-Si-B alloys should consist of a continuous Mo phase with homogeneously dispersed intermetallic particles. While a continuous Mo phase was favorable for ductility and damage tolerance, the intermetallic phases serve as high-temperature reinforcements, enhancing oxidation and creep resistance. Further detailed studies to explore the relationship between microstructure and mechanical properties are planned for future work.

## Conclusion

So far, the development of additive manufacturing processes for new materials has primarily relied on empirical testing and iterative data collection and analysis. In this work, the Mo-9Si-8B (at.%) alloy was successfully processed via PBF-EB for the first time, and the processing window was rapidly developed through strategies that combine high-throughput thermal modeling for melt pool dimension prediction with in situ electron-optical imaging (ELO). The consolidation and persistence boundaries were identified through the predicted melt pool dimensions to guide process parameter selection. In situ ELO monitoring further enabled the immediate identification and classification of sample surfaces, eliminating the necessity of conventional sample preparation and analysis. A qualitative comparison of both approaches demonstrated a high degree of agreement, enabling accurate processing window determination while significantly reducing experimental effort. According to the established processing window, high-density bulk Mo-9Si-8B alloy consisting of Mo_SS_, Mo_5_SiB_2_, and Mo_3_Si phases, was successfully fabricated at varying lateral velocities with constant area energy. The Mo_SS_ phase exhibits dendritic structures with fine Mo_5_SiB_2_ and Mo_3_Si interdendritic phases. This novel strategy significantly reduced the effort required to establish processing windows, enhancing the viability of establishing stable processing conditions for new materials via PBF-EB. 

## Data Availability

Data will be made available on request.
